# Melatonin Promotes Ubiquitination of Phosphorylated Pro-Apoptotic Protein Bcl-2-Interacting Mediator of Cell Death-Extra Long (Bim_EL_) in Porcine Granulosa Cells

**DOI:** 10.3390/ijms19113431

**Published:** 2018-11-01

**Authors:** Yingzheng Wang, Shenming Zeng

**Affiliations:** College of Animal Science and Technology, Yangzhou University, Yangzhou 225009, China; yingzhengwang1@163.com

**Keywords:** melatonin, apoptosis, Bim_EL_, ubiquitination, degradation

## Abstract

Melatonin (*N*-acetyl-5-methoxytryptamine) is found in ovarian follicular fluid, and its concentration is closely related to follicular health status. Nevertheless, the molecular mechanisms underlying melatonin function in follicles are uncertain. In this study, melatonin concentration was measured in porcine follicular fluid at different stages of health. The melatonin concentration decreased as the follicles underwent atresia, suggesting that melatonin may participate in the maintenance of follicular health. The molecular pathway through which melatonin may regulate follicular development was further investigated. The pro-apoptotic protein Bim_EL_ (Bcl-2-interacting mediator of cell death-Extra Long), a key protein controlling granulosa cell apoptosis during follicular atresia, was selected as the target molecule. Bim_EL_ was downregulated when porcine granulosa cells were cultured in medium containing 10^−9^ M melatonin and isolated cumulus oocyte complexes (COCs) or follicle stimulating hormone (FSH). Interestingly, ERK-mediated phosphorylation was a prerequisite for the melatonin-induced decline in Bim_EL_, and melatonin only promoted the ubiquitination of phosphorylated Bim_EL_, and did not affect the activities of the lysosome or the proteasome. Moreover, the melatonin-induced downregulation of Bim_EL_ was independent of its receptor and its antioxidant properties. In conclusion, melatonin may maintain follicular health by inducing Bim_EL_ ubiquitination to inhibit the apoptosis of granulosa cells.

## 1. Introduction

During mammalian follicular development, only a limited number of follicles are selected to ovulate, with the remainder undergoing atresia at different stages. Follicle atresia is triggered by the apoptosis of granulosa cells, and several apoptotic-signaling molecules, such as hormones/growth factors/cytokines, the death ligand-receptor system, and B cell lymphoma/leukemia 2 (Bcl-2) family members, are involved in this process [1,2,3,4]. The Bcl-2 family, which includes both anti-apoptotic (Bcl-2, B cell lymphoma/leukemia X (Bcl-X)) and pro-apoptotic (Bcl-2 interacting domain (Bid), Bim, Bax, Bak) proteins, are key regulators of apoptosis, and members of the Bcl-2 protein family play pivotal roles in follicular growth and atresia [2,5,6].

Bim (Bcl-2 interacting mediator of cell death), a BH3-only family member, binds with high affinity to anti-apoptotic Bcl-2 family members and regulates apoptotic signaling through Bax and Bak [7]. Moreover, Bim can be phosphorylated by several MAP kinases to regulate its activity [8]. Bim_EL_ is the predominant isoform of Bim in several mammalian tissues, as determined using Western blotting analysis [9]. Our previous studies showed that Bim_EL_ participates in porcine follicular atresia through regulating granulosa cell apoptosis, and FSH and GDF9 regulate its expression [9,10]. In addition to its transcriptional regulation, the post-translational modification of Bim_EL_ is also important for its function [11,12]. However, the regulation of Bim_EL_ modification during mammalian follicular development is still not clearly defined.

Melatonin (*N*-acetyl-5-methoxytryptamine) can be synthesized from tryptophan in different cells, tissues, and organs, and mainly for local utilization (autocrine and paracrine actions). However, circulating melatonin is largely secreted rhythmically by the pineal gland [13,14]. Melatonin is found in follicular fluid and its concentration is higher than that in blood [15,16]. Granulosa cells, cumulus cells, and oocyte have been reported as able to synthesize melatonin [17,18,19] and melatonin receptors (MT1, MT2) expressed in human granulosa cells [20]. So far, the function of melatonin as an antioxidant and free radical scavenger in follicular development is well-established [21,22]. Moreover, melatonin has been reported to exhibit anti-apoptotic effects in different cells [23,24]. However, previous studies indicated that melatonin could also induce apoptosis under the regulation of Bim in many different cancer cell lines, including human hepatocellular carcinoma cells, breast carcinoma MDA-MB231 cells, and renal cancer Caki cells [11,25,26]. These findings imply that Bim_EL_ has diverse functions in different cell types. To date, the exact mechanism of melatonin on follicular development is still not clear and requires further study. In this study, we explored the relationship between melatonin and Bim_EL_ in porcine follicle granulosa cells.

## 2. Results

### 2.1. Melatonin Downregulates Bim_EL_ in Porcine Granulosa Cells

**Figure 1:**
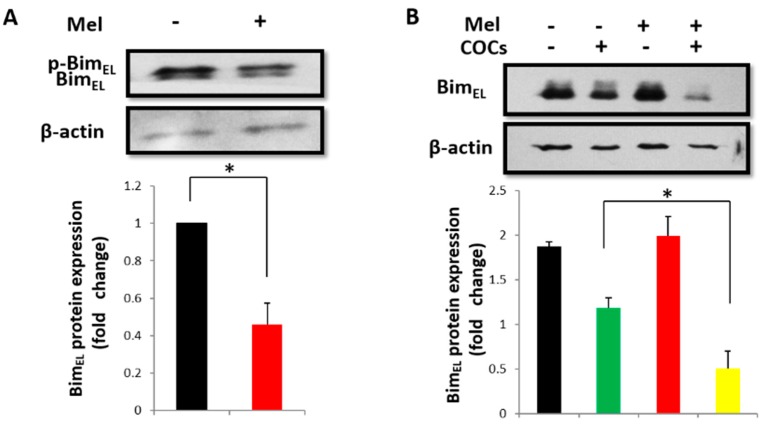

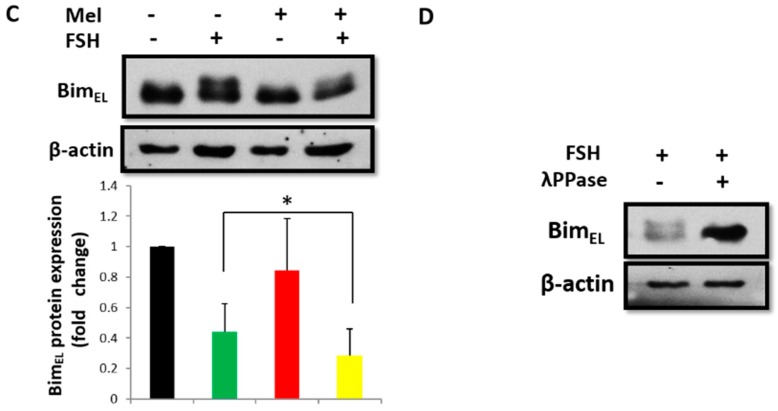
Melatonin downregulates the Bim_EL_ protein in porcine cumulus granulosa cells and primary granulosa cells treated with COCs or FSH. (**A**) Bim_EL_ level decreased in porcine cumulus granulosa cells after COCs were treated with 10^−9^ M melatonin (Mel) for 42–44 h. (**B**) Bim_EL_ level decreased in porcine primary granulosa cells treated with 10^−9^ M melatonin in the presence of COCs for 24 h. (**C**) Bim_EL_ level decreased in porcine primary granulosa cells treated with 10^−9^ M melatonin and 0.01 IU/mL FSH for 24 h. (**D**) Western blot analyses of Bim_EL_ phosphorylation after porcine primary granulosa cells were treated with 0.01 IU/mL FSH for 24 h. Whole-cell lysates were obtained and incubated with or without λ phosphatase (λ PPase). Values are expressed as the means ± S.D of three separate experiments. * *p* < 0.05.

The Bim_EL_ protein in the surrounding cumulus granulosa cells decreased significantly after porcine COCs were treated with 10^−9^ M melatonin for 42–44 h (Figure 1A). However, there was no change in the Bim_EL_ level in primary granulosa cells after treatment with 10^−9^ M melatonin for 24 h (Figure 1B). Interestingly, melatonin significantly decreased the Bim_EL_ level when primary granulosa cells were cocultured with COCs (Figure 1B). Similar results were obtained after granulosa cells were treated with FSH (Figure 1C). Furthermore, COCs exposure and FSH treatment resulted in a Bim_EL_ protein mobility shift (Figure 1A–C), and this shift was inhibited when the lysates from FSH-treated primary granulosa cells were incubated with λ phosphatase (Figure 1D), indicating that the observed Bim_EL_ mobility shift was caused by its phosphorylation. These results also indicated that COCs or FSH could induce Bim_EL_ phosphorylation in granulosa cells. Thus, these data suggest that the function of melatonin in the downregulation of Bim_EL_ may depend on its phosphorylation level.

### 2.2. Melatonin Downregulates Bim_EL_ Depending on ERK Activation and Bim_EL_ Phosphorylation

**Figure 2:**
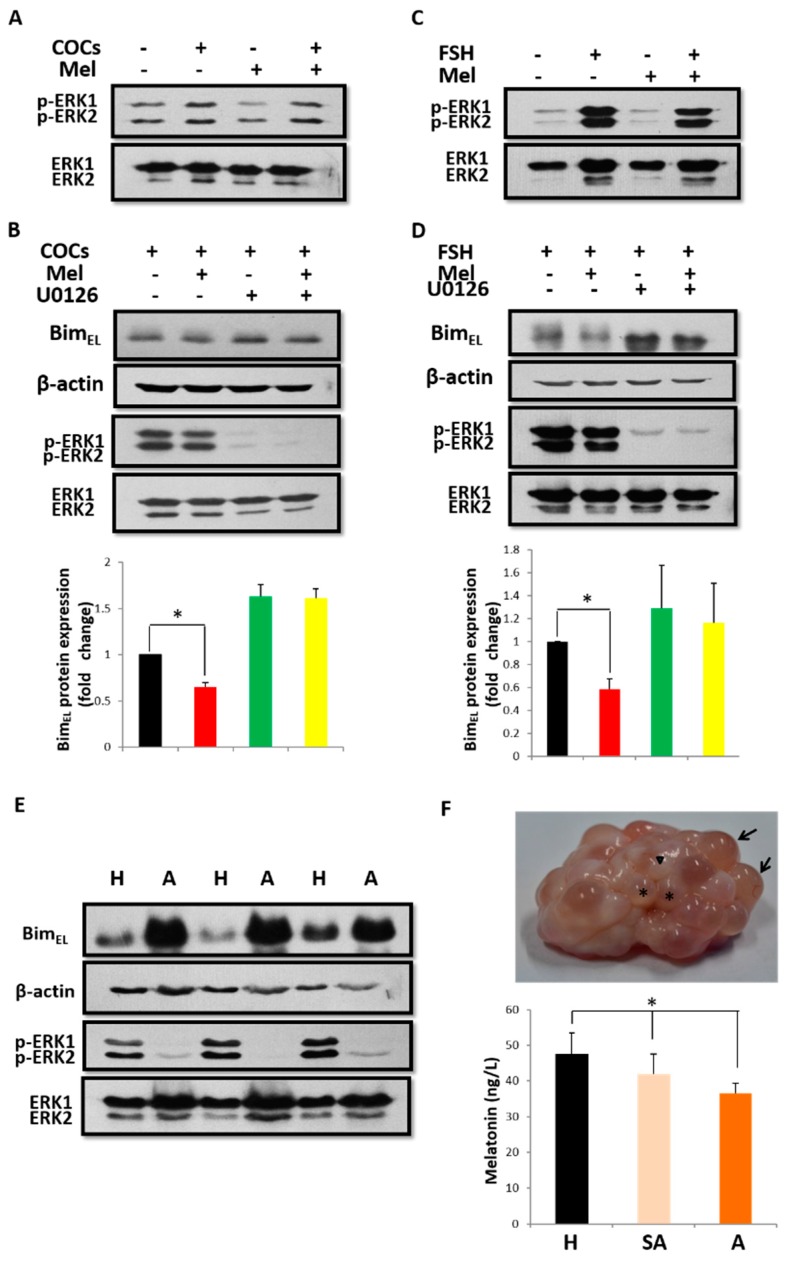
Melatonin downregulates Bim_EL_ protein by COCs or FSH-mediated, activating the ERK pathway in porcine primary granulosa cells. (**A**) Phosphorylated ERK level increased in porcine primary granulosa cells treated with 10^−9^ M melatonin (Mel) in the presence of COCs for 24 h. (**B**) Inhibition of ERK phosphorylation by 20 μM U0126 prevented the decrease in Bim_EL_ level induced by melatonin and COCs, coinciding with the decrease in phosphorylated ERK. (**C**) phosphorylated ERK level increased in porcine primary granulosa cells treated with 10^−9^ M melatonin in the presence of FSH for 24 h. (**D**) Inhibition of ERK phosphorylation by 20 μM U0126 prevented the decrease in Bim_EL_ level induced by melatonin and FSH, coinciding with the decrease in phosphorylated ERK. (**E**) There was an inverse relationship between levels of Bim_EL_ and phosphorylated ERK in porcine granulosa cells from healthy or atretic follicles. (**F**) Melatonin concentration decreased in follicles with progressive atresia. H, healthy follicles (arrows); SA, slightly atretic follicle (arrowhead); A, atretic follicles (asterisks). Data are representative of three independent experiments. Values are expressed as the means ± S.D. of three separate experiments. * *p* < 0.05.

Phosphorylation of Bim_EL_ by extracellular signal-regulated kinase 1/2 (ERK1/2) has been shown to induce the degradation of Bim_EL_ [27], so we first determined the status of ERK activation. As shown in Figure 2A,C, ERK1/2 in granulosa cells was activated after treatment with COCs or FSH, but it was not induced by melatonin, revealing that melatonin could not directly promote the degradation of Bim_EL_ by phosphorylation. To determine the precise role of melatonin in this process, primary granulosa cells cultured in the presence of COCs or FSH were pretreated with U0126, an MEK1/2 inhibitor, before melatonin treatment. As expected, U0126 abolished the induction of Bim_EL_ phosphorylation by COCs or FSH in parallel with the abrogation of Bim_EL_ downregulation caused by melatonin (Figure 2B,D). These results confirmed that the phosphorylation of Bim_EL_ induced by ERK was a prerequisite for Bim_EL_ reduction induced by melatonin. To further confirm whether this phenomenon exists in follicles in vivo, the lysates from granulosa cells obtained from healthy or atretic follicles were subjected to SDS-PAGE to detect ERK activation and Bim_EL_ expression. As shown in Figure 2E, the level of activated ERK1/2 was higher, whereas the Bim_EL_ level was lower, in granulosa cells of healthy follicles compared to atretic follicles. Furthermore, melatonin concentration decreased with the atresia of porcine ovarian follicles. The concentrations of melatonin in healthy, slightly atretic, and atretic follicles were 47.47 ± 6.03 ng/L, 41.97 ± 5.66 ng/L, and 36.50 ± 2.84 ng/L, respectively, and the difference between healthy follicles and slightly atretic or atretic ones was significant (*p* < 0.05, Figure 2F). These results suggest that ERK activation is responsible for the induction of Bim_EL_ phosphorylation by COCs or FSH, and it promotes melatonin-induced Bim_EL_ downregulation in porcine granulosa cells. This process is likely to play a vital role in maintaining follicle health.

### 2.3. Post-Translational Pathway Is Involved in Melatonin-Induced Downregulation of Bim_EL_

**Figure 3:**
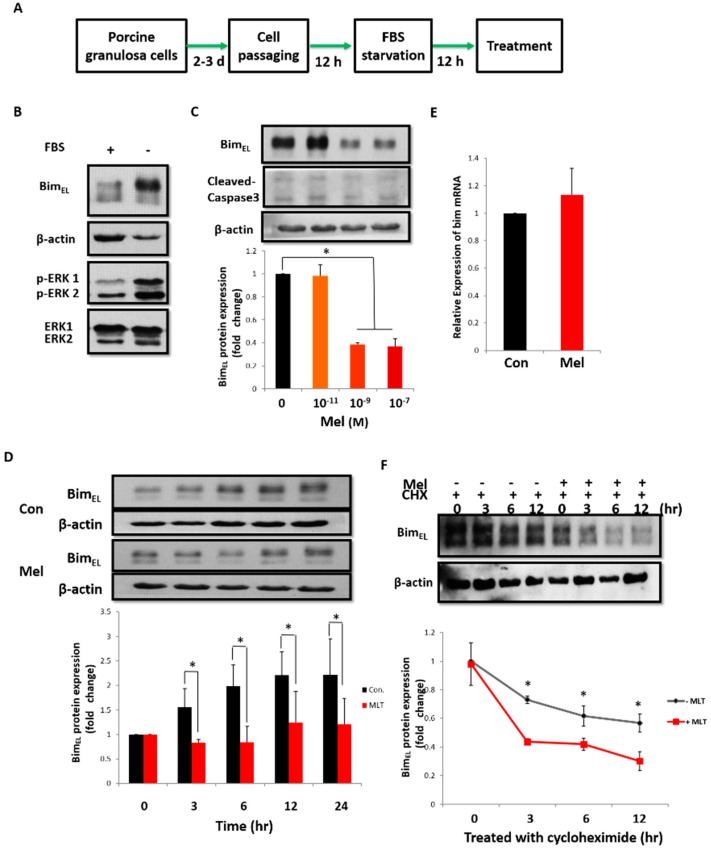
Melatonin decreases Bim_EL_ protein in porcine adherent granulosa cells. (**A**) Experimental protocol. Porcine primary granulosa cells were cultured for two to three days, passaged, and cultured for an additional 12 h, and then incubated with serum-free medium for 12 h. Thereafter, different treatments were performed. (**B**) Levels of phosphorylated Bim_EL_ and ERK increased in porcine adherent granulosa cells after culturing in serum-free medium for 12 h. (**C**) Bim_EL_ decreased in porcine adherent granulosa cells 24 h after melatonin treatment. (**D**) Bim_EL_ decreased in porcine adherent granulosa cells within 3 h of melatonin treatment. (**E**) Melatonin did not affect *bim* mRNA expression in porcine adherent granulosa cells. (**F**) Melatonin accelerated Bim_EL_ degradation in porcine adherent granulosa cells treated with cycloheximide (CHX). Values are expressed as the means ± S.D. of three separate experiments. * *p* < 0.05.

The molecular mechanism of melatonin-induced downregulation of Bim_EL_ was systemically investigated using porcine adherent granulosa cells with the experimental protocol shown in Figure 3A. After 12 h of serum withdrawal, a significant increase in phosphorylated Bim_EL_ was observed (Figure 3B), accompanied by a robust activation of ERK1/2, which was similar to that in primary granulosa cells treated with COCs or FSH. To determine whether melatonin could downregulate the Bim_EL_ protein in porcine adherent granulosa cells, cells were treated with melatonin at different concentrations (0, 10^−11^, 10^−9^, 10^−7^ M) for 24 h. As shown in Figure 3C, the levels of Bim_EL_ and Cleaved Caspase3 significantly decreased after 10^−9^ M melatonin treatment, and this effect was evident within 3 h after treatment (Figure 3D).

Because the Bim_EL_ protein expression level can be regulated by transcriptional and post-translational pathways, our next experiments aimed to determine the mechanism responsible for this change. As shown in Figure 3E, there was no difference in the mRNA expression of *bim* 3 h post melatonin treatment compared to the control group. Therefore, we hypothesized that the downregulation of Bim_EL_ was controlled by post-translational modifications. To address this, porcine adherent granulosa cells were incubated with cycloheximide (CHX) alone or co-treated with CHX and melatonin for indicated time periods (Figure 3F). The combination of CHX and melatonin induced a rapid decrease in the Bim_EL_ level within 3 h after treatment compared with CHX alone. These results indicate that the melatonin-mediated Bim_EL_ decline is regulated at the post-translational level, and Bim_EL_ is actively degraded.

### 2.4. Melatonin Promotes Bim_EL_ Ubiquitination

**Figure 4:**
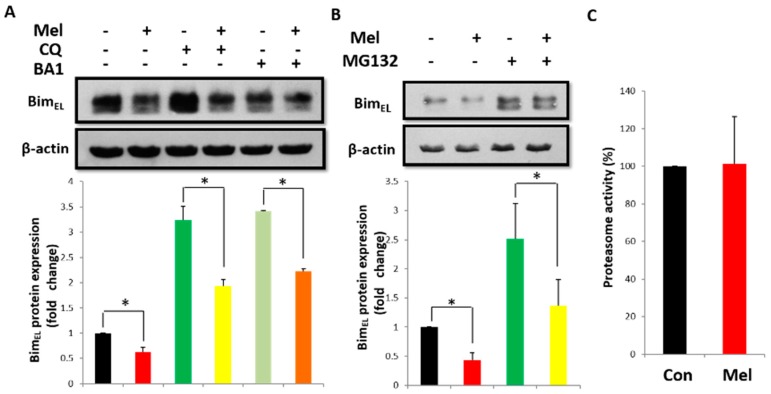
Neither lysosomal activity nor proteasomal activity is involved in downregulation of Bim_EL_ induced by melatonin. (**A**) Lysosomal inhibitor chloroquine (CQ) and Bafilomycin A1 (BA1) did not block the downregulation of Bim_EL_ caused by melatonin in porcine adherent granulosa cells. (**B**) Proteasomal inhibitor MG132 did not affect the downregulation of Bim_EL_ in porcine adherent granulosa cells. (**C**) Melatonin did not affect proteasomal activity. Values are expressed as the means ± S.D. of three separate experiments. * *p* < 0.05.

Proteasomes and lysosomes comprise two major intracellular proteolytic systems in mammalian cells. Hence, we investigated whether Bim_EL_ was increasingly degraded by lysosomes or proteasomes following melatonin treatment. First, porcine adherent granulosa cells were treated with melatonin in the presence of a potent inhibitor of the vacuolar type H^+^-ATPase, Bafilomycin A1, or chloroquine, which accumulated in lysosomes and raised the intralysosomal pH value. The inhibitors induced the accumulation of LC3-II (data not shown) to manifest its inhibition of lysosomal proteolysis. As shown in Figure 4A, Bafilomycin A1 and chloroquine failed to block Bim_EL_ downregulation by melatonin, indicating that the proteasomal pathway was responsible for Bim_EL_ degradation. Protein phosphorylation is required to prepare Bim for ubiquitination and proteasomal degradation. Porcine adherent granulosa cells were treated with melatonin in the presence of the proteasome inhibitor MG132 for 3 h. However, MG132 could also not counteract the Bim_EL_ repression by melatonin (Figure 4B). To confirm whether melatonin could influence the proteasomal activity in granulosa cells, we measured chymotrypsin-like activity using a commercially-available proteasome 20S assay kit. Melatonin did not change the proteasomal activity in granulosa cells (Figure 4C). These results indicate that neither lysosomes nor proteasomes directly participate in Bim_EL_ reduction by melatonin.

**Figure 5:**
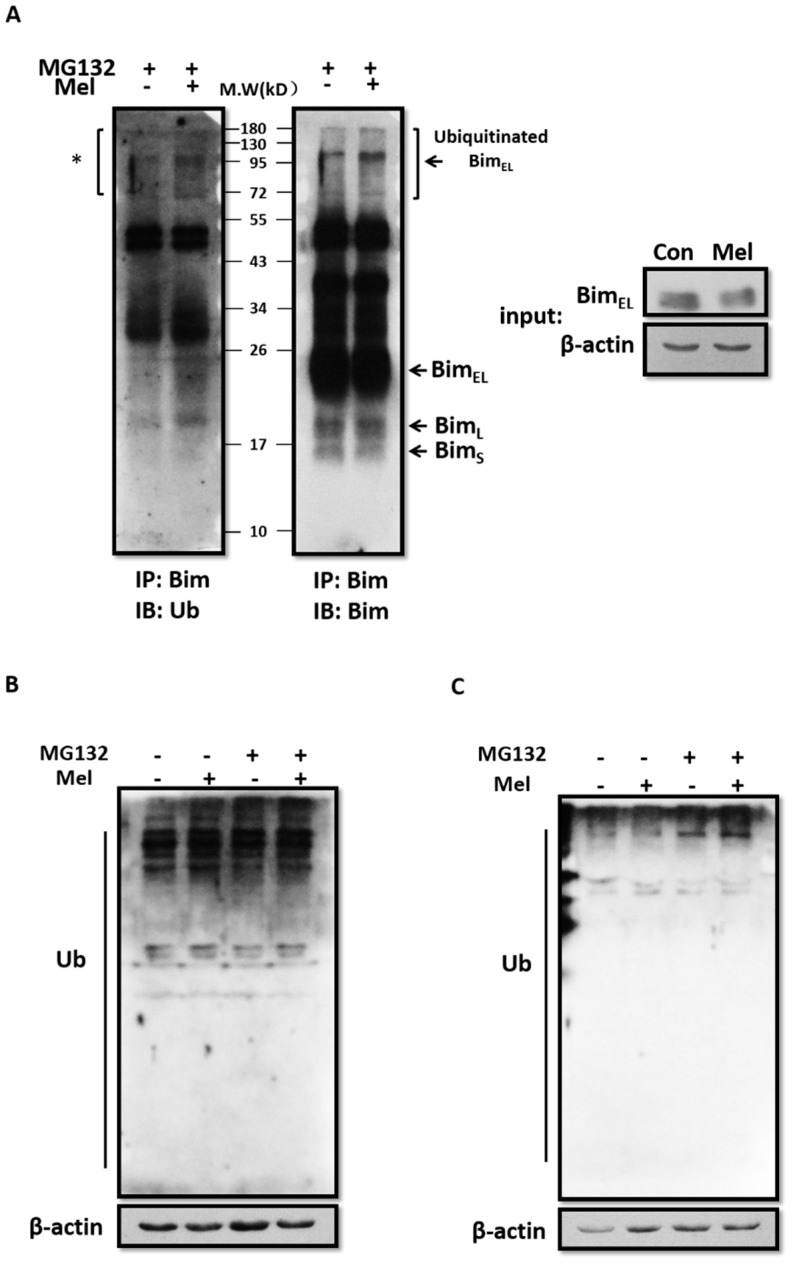
Melatonin induces the ubiquitination of Bim_EL_ and other proteins. (**A**) Bim_EL_ ubiquitination increased in porcine adherent granulosa cells treated with 10^−9^ M melatonin in the presence of 5 μM MG132 for 3 h. Bim was immunoprecipitated, and Western blotting was performed using anti-Bim and ubiquitin (Ub) antibodies. The asterisk indicates ubiquitinated Bim_EL_ appearing as a smear of bands with higher molecular weights. The level of protein ubiquitination increased in porcine adherent granulosa cells (**B**) and porcine primary granulosa cells (**C**) treated with 10^−9^ M melatonin in the presence of MG132 (5 μM) for 3 h. Western blotting was performed with an anti-ubiquitin antibody. Data are representative of three independent experiments.

It has been shown that activation of the ERK pathway promotes the phosphorylation of Bim_EL_, which serves to mark Bim_EL_ for ubiquitination [28]. Immunoprecipitation of Bim_EL_, followed by immunoblot analysis using an anti-ubiquitin antibody, demonstrated that melatonin enhanced the level of Bim_EL_ poly-ubiquitination in porcine adherent granulosa cells supplemented with MG132 (Figure 5A, left lane). We found that the level of ubiquitinated Bim_EL_ increased, whereas levels of Bim_EL_, Bim_L_, and Bim_S_ decreased after melatonin treatment (Figure 5B, right lane). Western blot analysis of whole-cell lysates showed that melatonin also elevated the overall level of ubiquitination in both porcine primary and adherent granulosa cells (Figure 5B,C). Taken together, our data suggest that melatonin activates the ubiquitination of Bim_EL_.

### 2.5. Reduction of Bim_EL_ by Melatonin Does Not Associate with Its Receptor or Antioxidant Properties

**Figure 6:**
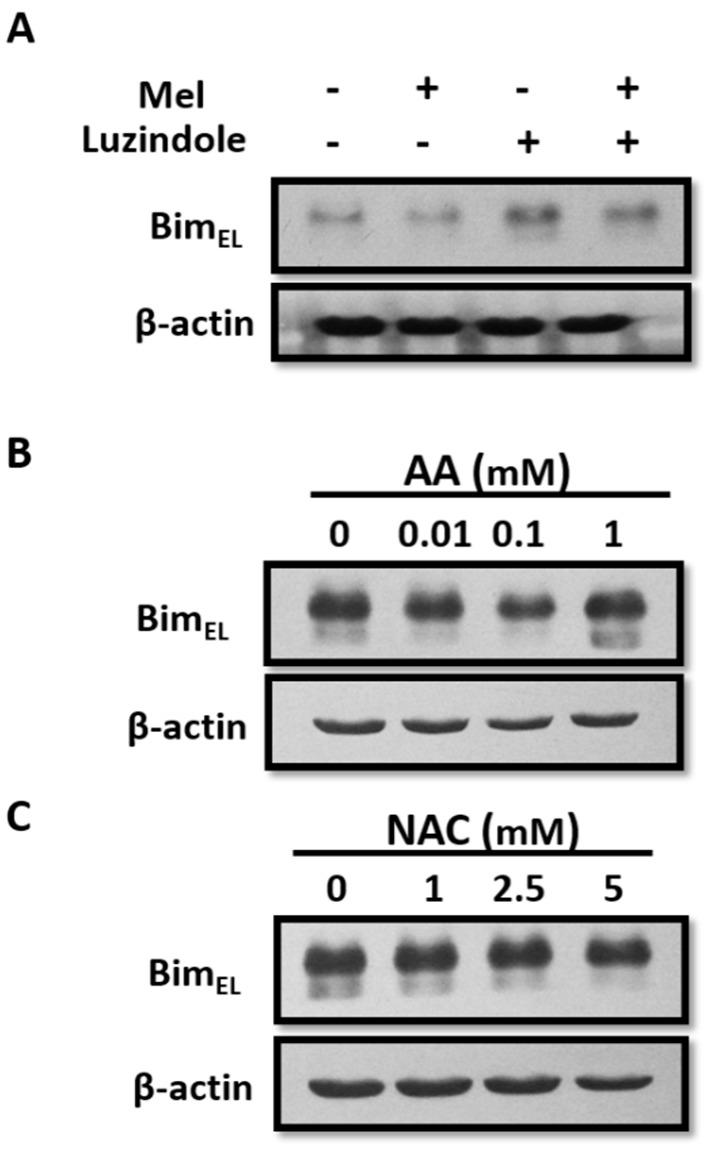
Melatonin receptors and melatonin antioxidant properties are not involved in Bim_EL_ downregulation. (**A**) Bim_EL_ degradation caused by melatonin was not affected by luzidole, a melatonin receptor antagonist, in porcine adherent granulosa cells. (**B**,**C**) Antioxidants AA and NAC had no effects on the Bim_EL_ level in porcine adherent granulosa cells. Data are representative of three independent experiments.

To classify the underlying mechanisms of the melatonin-induced decrease in Bim_EL_, we investigated the potential role of melatonin receptors because various physiological effects of melatonin can be mediated by its two G-protein-coupled MT1 and MT2 receptors. Porcine adherent granulosa cells were treated with melatonin in the presence of luzindole, a melatonin receptor antagonist, and then Bim_EL_ protein was examined. As shown in Figure 6A, luzindole failed to block the Bim_EL_ downregulation by melatonin. In addition, melatonin is known to be a powerful antioxidant. To determine the relevance of antioxidant activity on Bim_EL_ degradation, we tested the effects of two other antioxidant reagents, *N*-acetylcysteine (NAC) and ascorbic acid (AA), on the Bim_EL_ protein in porcine adherent granulosa cells. The western blot showed no change in the level of Bim_EL_ after treatment compared to the control (Figure 6B,C). Based on these data, we conclude that melatonin-mediated Bim_EL_ downregulation is independent of the melatonin receptor-mediated pathway or its antioxidant function.

## 3. Discussion

Melatonin plays a pivotal role in female reproduction, including puberty, ovarian follicle growth, ovulation, and luteinization [29,30,31]. Several studies have demonstrated that melatonin has beneficial effects on oocyte maturation and subsequent embryo development in many species, such as mice [32], cattle [33], and pigs [15]. Except for its antioxidant properties, however, the role of melatonin in female reproduction remains largely unknown. Throughout the reproductive life span, more than 99% of germ cells are eliminated from the ovary through follicular atresia, and granulosa cells play a major role in this process. It is well-established that the initial step of follicular atresia is granulosa cell apoptosis [34]. Our previous study showed that the pro-apoptotic protein Bim_EL_ plays an important role in porcine granulosa cell apoptosis [9]. In this study, we demonstrated that melatonin decreases the Bim_EL_ protein via inducing its ubiquitination through the post-translational pathway in porcine granulosa cells.

It has been shown that the supplementation of porcine maturation medium with 10^−9^ M melatonin is beneficial for in vitro maturation (IVM) of porcine oocytes and subsequent embryo development [15]. Moreover, our previous study showed that Bim_EL_-mediated apoptosis in cumulus cells accelerates oocyte aging and degeneration [35]. In this study, we showed that melatonin decreased the Bim_EL_ protein in cumulus granulosa cells during IVM. Granulosa cells play an important role in supporting oocyte maturation, which prompted us to focus on the effects of melatonin on Bim_EL_ protein expression in porcine primary granulosa cells. However, melatonin treatment alone failed to decrease the Bim_EL_ level. Conversely, a combination of melatonin with COCs or FSH downregulated the Bim_EL_ protein, although melatonin can induce *bim* expression in several cancer cell lines [11,25,26]. Thus, these results suggested that the mechanism of regulating *bim* expression may be different between cancer cells and normal tissue cells or these contradictory results are from using different melatonin concentrations.

Our results demonstrated that ERK1/2 was activated in granulosa cells by COCs and FSH rather than by melatonin. These results were consistent with those of a previous report by Baumgarten et al., who found that FSH could activate the ERK pathway in human cumulus granulosa cells [36]. Oocyte-secreted factors such as GDF9 also activate ERK in human granulosa cells. A recent study reported that melatonin activated ERK1/2 in HEK293 cells in a concentration-dependent manner after 5 min of treatment [37]. Moreover, the activation of ERK1/2 induced by melatonin antagonized UVB-induced apoptosis in U937 cells [38]. Thus, melatonin appears to activate ERK1/2 in different cells. ERK1/2 kinase was responsible for Bim_EL_ phosphorylation because the MEK1/2 inhibitor U0126 blocked COC- and FSH-induced Bim_EL_ phosphorylation. However, U0126 can partially inhibit MEK5 and ERK5 activation [39]; thus, the phosphorylation of Bim_EL_ may also be partially due to ERK5 activation. Moreover, accumulating data indicate that multiple phosphorylated isoforms of Bim_EL_ are regulated by different kinase pathways, including ERK, JNK, and p38 MAP kinases, which may result in different apoptotic end-points [8]. For example, UV-mediated JNK activation results in the phosphorylation of Bim_EL_ on Thr-112, potentiating its apoptotic activity [40], and sodium arsenite-induced apoptosis causes the phosphorylation of Bim_EL_ at Ser-65 by p38 in PC12 cells [41]. In contrast, phosphorylation by ERK on Ser-55/65/73 targeted Bim_EL_ for degradation via the ubiquitin-proteasome pathway and promoted cell survival [28]. In the present study, melatonin-induced downregulation of Bim_EL_ was abolished by U0126, indicating that Bim_EL_ phosphorylation by ERK was essential for this process; however, the specific phosphorylated sites in Bim_EL_ induced by COCs or FSH should be confirmed in a further study.

In this study, we observed correlations among ERK1/2 activation, the Bim_EL_ level, and follicle health status. An inverse relationship between ERK activation and Bim_EL_ level was observed in porcine follicles, with healthy follicles displaying a higher level of phosphorylated ERK than atretic ones. Similarly, higher levels of phosphorylated ERK in dominant follicles were detected compared with subordinate follicles in sheep [42]. These data suggest that ERK activation plays an important role, not only in melatonin-induced downregulation of Bim_EL_, but also in the process of dominant follicle selection.

We showed that serum starvation of porcine adherent granulosa cells significantly activated ERK and enhanced Bim_EL_ phosphorylation, which provided an ideal model to mimic the effects of COCs and FSH in primary granulosa cells. The same phenomenon of ERK activation by serum starvation also exists in human colon carcinoma cells [43]. Following the activation of ERK, melatonin downregulated the Bim_EL_ protein and Cleaved Caspase 3 level in porcine adherent granulosa cells (Figure 3C). It is well-established that gene transcription, mRNA stability, and post-translational modifications regulate the Bim_EL_ level by different stimuli [8,44,45]. The transcriptional factors FOXO3a, Runx3, E2F1, c-Jun, and SP1 have been shown to regulate *bim* transcription. During follicular development, FSH regulates Bim_EL_ expression through FoxO3a in granulosa cells [9]. It was demonstrated that melatonin induces the expression of transcription factors of Sp1 and E2F1, coinciding with the induction of Bim_EL_ in renal cancer Caki cells [11]. However, we did not detect an obvious mRNA change of *bim* in porcine granulosa cells by real-time PCR after melatonin treatment (Figure 3E). Moreover, a significant decline of Bim_EL_ was observed when granulosa cells were treated with melatonin in the presence of CHX (Figure 3F), implying that the downregulation of Bim_EL_ in granulosa cells by melatonin was closely associated with its post-translational modification.

Proteasomes or lysosomes play imperative roles in controlling intracellular protein quantity and quality [46]. Choi et al. reported that melatonin increased the degradation of TGFBlp via activating autophagy and counteracted the inhibition of autophagy by Bafilomycin A1 in corneal fibroblasts [47]. Previous studies have shown that melatonin can inhibit proteasome activity [48]. Nevertheless, neither a lysosomal-degradation inhibitor nor a proteasomal inhibitor could inhibit the decline in Bim_EL_ induced by melatonin in our study. Unexpectedly, melatonin markedly increased the ubiquitination of Bim_EL_. According to our results, melatonin may also have induced the ubiquitination of other proteins in granulosa cells. Therefore, melatonin decreased the Bim_EL_ level by increasing its ubiquitination. Although melatonin was previously regarded as an inhibitor of the ubiquitin-proteasome system [49], our contradictory results may be attributed to different cells with different experimental conditions. Park et al. reported that melatonin could increase the level of Bim_EL_ by inhibiting the activity of proteasomes to induce apoptosis of human renal cancer cells [11]. In their experiment, cancer cells were treated with melatonin at the concentration from 0.1 to 1 mM, but the concentration was just 1 nM in our experiment. Above all, according to previous studies, melatonin presented toxicity to the oocyte during maturation when its concentration reached at 10^−5^ M [15]. In addition, the effect of melatonin on proteasome activity may be different between cancer cells and granulosa cells. It has been shown that mitochondrial-associated Bcl-2 family proteins, including Bax, Bcl-2, and Bim, are regulated through ubiquitin/proteasomal degradation during apoptosis [50]. Wan et al. reported that APCCdc20 acts as an E3 ubiquitin ligase to promote Bim ubiquitination and destruction [51]. Additional studies are required to delineate the precise mechanisms involved in melatonin-induced protein ubiquitination in granulosa cells.

Generally, melatonin participates in various physiological processes via its receptors or antioxidant properties [52,53,54]. However, our results showed that melatonin downregulated Bim_EL_ through neither a receptor-mediated nor antioxidant pathway. Therefore, the detailed mechanism of Bim_EL_ downregulation by melatonin in granulosa cells waits further investigation.

**Figure 7:**
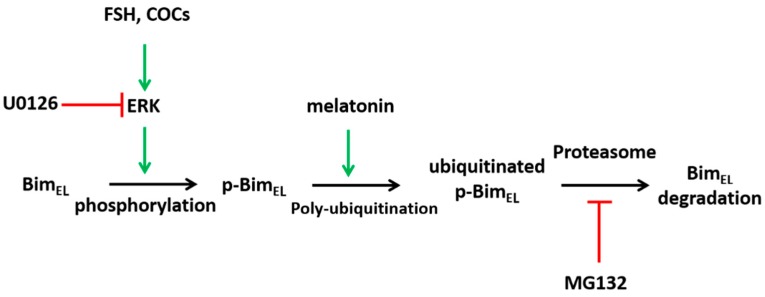
Diagram of Bim_EL_ degradation as regulated by melatonin in porcine granulosa cells.

A hypothetical model of Bim_EL_ regulation by melatonin in granulosa cells is presented in Figure 7. Although there are some details that need to be further pursued, our results demonstrate that melatonin downregulates phosphorylated Bim_EL_ via a ubiquitin-proteasome pathway in porcine granulosa cells to maintain follicle health. Future studies should investigate how melatonin precisely regulates protein ubiquitination. 

## 4. Materials and Methods

All chemicals used in this study were purchased from Sigma Chemical Co. (St. Louis, MO, USA) unless otherwise specified.

### 4.1. Classification of Follicles and Measurement of Melatonin in Porcine Follicular Fluid

Follicles were classified as healthy or atretic according to previously established morphological criteria [53]. In brief, healthy follicles had vascularized theca interna and clear amber follicular fluid with no debris. The slightly atretic and atretic follicles had gray theca interna and flocculent follicular fluid of different degrees. The concentration of melatonin in follicular fluid was assessed using the porcine melatonin ELISA kit (Shanghai MLBIO Biotechnology Co., Ltd., Shanghai, China) with a measurement range for melatonin from 1.5–65 ng/L.

### 4.2. In Vitro Maturation of Oocytes

In vitro maturation (IVM) of oocytes was performed as previously described [35]. In brief, porcine ovaries were collected at a local abattoir and transported to the laboratory within 2–3 h after collection. Cumulus oocyte complexes (COCs) were aspirated, and those with several layers of unexpanded cumulus cells were cultured in maturation medium for 42–44 h. The maturation medium was Tissue Culture Medium 199 with Earle’s salts (TCM199; Invitrogen, Carlsbad, CA, USA) supplemented with 10% porcine follicular fluid, 10 IU/mL hCG (Chorulon, Intervet Australia Pty Ltd., Victoria, Australia), 10 IU/mL eCG (Folligon, Intervet Australia Pty Ltd.), 10 ng/mL EGF, 0.6 mM cysteine, 75 mg/L penicillin, and 50 mg/L streptomycin. After IVM, cumulus cells were separated from oocytes and lysates were used for Western blotting.

### 4.3. Cell Culture and Treatment

Porcine primary or adherent granulosa cells were cultured as previously described [9]. In brief, porcine ovaries were collected at a local abattoir and transported to the laboratory within 2–3 h after collection. Ovaries were washed thrice with sterile 0.9% saline (37 °C) containing 100 IU/L penicillin and 100 mg/L streptomycin. Granulosa cells were then isolated by puncturing healthy follicles (2–5 mm in diameter) with a 25-gauge hypodermic needle and gently washing thrice with DMEM/F12 supplemented with 1% fetal bovine serum, 100 IU/L penicillin, and 100 mg/L streptomycin. Primary granulosa cells were selected under a microscope for different treatments according to the experimental design. In addition, granulosa cells were isolated by puncturing healthy follicles (2–5 mm in diameter) with a 25-gauge hypodermic needle and gently washing thrice with DMEM containing 10% fetal bovine serum, 100 IU/L penicillin and 100 mg/L streptomycin. Cells were then incubated at 37 °C in a humidified atmosphere of 5% CO_2_/95% air for 24 h. The cells were passaged upon reaching confluence. The culture medium was replaced with DMEM containing 100 IU/L penicillin and 100 mg/L streptomycin at 12 h after passaging, and the cells were cultured for an additional 12 h. Thereafter, the cells were treated with melatonin and/or other compounds for the indicated time periods. The cells were pretreated with 10 μM LY294002, 1 μM cycloheximide, 10 μM choloroquine, 100 nM Bafilomycin A1, or 5 μM MG132 at 1 h before melatonin treatment.

### 4.4. Western Blotting

The cells were lysed in Laemmli sample buffer (Bio-Rad, Hercules, CA, USA). An equal amount of protein was separated by sodium dodecyl sulfate polyacrylamide gel electrophoresis (SDS-PAGE; 12% acrylamide gel), and proteins were transferred to nitrocellulose membranes (Millipore, Billerica, MA, USA). After blocking with 5% non-fat milk Tris-buffered saline containing 0.1% Tween-20 (TBST), the membranes were incubated with primary antibodies overnight at 4 °C. After washing with TBST, the membranes were incubated with the appropriate secondary antibodies conjugated to horseradish peroxidase at a dilution of 1:2000 for 1 h. The protein bands were visualized using an enhanced chemiluminescence detection system (Applygen Technologies Inc., Beijing, China). The western blotting images were processed using Image J software (National Institutes of Health, Bethesda, MD, USA).

### 4.5. Lambda Phosphatase Treatment

Whole-cell extracts (30 μg) were incubated with or without 1 μL of lambda protein phosphatase (400 U/μL; New England BioLabs, Ipswich, MA, USA) for 2 h at 30 °C. The samples were boiled for 5 min after adding 10 μL of 5× SDS sample buffer.

### 4.6. Proteasome Activity Assay

Chymotrypsin-like protease activity was measured using the Amplite™ Fluorometric Proteasome 20S Assay Kit (AAT Bioquest Inc., Sunnyvale, CA, USA) according to the manufacturer’s instructions. In brief, 100 μL of proteasome assay loading solution was added to each well after granulosa cells were treated with 10^−9^ M melatonin for 3 h and the tissue culture plate was then incubated at 37 °C for 2 h. The assay was performed by monitoring the fluorescence at 480 nm EX/520 nm EM.

### 4.7. Immunoprecipitation

To detect ubiquitinated Bim in whole-cell extracts, granulosa cells were homogenized in RIPA buffer containing a broad-spectrum protease inhibitor cocktail (Roche, Basel, Switzerland). The protein concentrations of the cell extracts were measured, and equal amounts of protein were incubated overnight at 4 °C with a polyclonal Bim antibody (Cell Signaling Technology, Beverly, MA, USA). The antibody-antigen complex was then incubated with protein A/G PLUS-agarose beads (Santa Cruz Biotechnology, Santa Cruz, CA, USA). The immunoprecipitated proteins were resolved by SDS-PAGE (12% acrylamide gel) and processed for Western blotting using specific antibodies to detect ubiquitin and Bim.

### 4.8. Real-Time quantitative PCR

A reverse transcription polymerase chain reaction was used to determine if melatonin regulated the mRNA expression of *bim*. First-strand cDNA synthesis was carried out with the SuperScript™ First-Strand Synthesis System for RT-PCR (Invitrogen) according to the manufacturer’s instructions. The real-time PCR primers for *bim* were: 5′-AGGCTGAACCCGCAGATA-3′ (forward) and 5′-GCATTAAATTCGTCTCCAATACG-3′ (reverse). The real-time PCR primers for β-actin were: 5′-ATCGTGCGGGACATAAG-3′ (forward) and 5′-CTCGTTGCCGATGGTGAT-3′ (reverse). The mRNA expression of *bim* was normalized to that of the endogenous control β-actin. Real-time PCR reactions were performed using the ABI 7900 Sequence Detection System (Applied Biosystems, Foster City, CA, USA).

### 4.9. Statistical Analysis

Data are presented as the means ± standard deviation (S.D.) of at least three independent replicates. Data were analyzed by one-way and two-way analysis of variance (ANOVA) and Duncan’s test using SAS software (SAS Institute, Cary, NC, USA). Differences were considered statistically significant at *p*-values < 0.05.
